# Oxytocin biases eye-gaze to dynamic and static social images and the eyes of fearful faces: associations with trait autism

**DOI:** 10.1038/s41398-020-0830-x

**Published:** 2020-05-12

**Authors:** Jiao Le, Juan Kou, Weihua Zhao, Meina Fu, Yingying Zhang, Benjamin Becker, Keith M. Kendrick

**Affiliations:** The Clinical Hospital of Chengdu Brain Science Institute, MOE Key Laboratory for Neuroinformation, University of Electronic Science and Technology of China, 611731 Chengdu, China

**Keywords:** Autism spectrum disorders, Molecular neuroscience, Autism spectrum disorders, Molecular neuroscience

## Abstract

A key functional effect of intranasal oxytocin with potential therapeutic relevance for autism-spectrum disorder is its reported facilitation of attention towards social stimuli, notably the eye region of faces. In the current randomized placebo-controlled within-subject experiment on 40 healthy males, we investigated the robustness of this facilitation of attention by intranasal oxytocin (24IU) towards social cues. Eye-tracking measures of preference for dynamic and static social vs. non-social stimuli were taken in four different paradigms where autistic individuals tend to exhibit reduced interest in social stimuli. Additionally, we investigated whether oxytocin increases attention towards the eyes relative to other salient face regions in an emotional face paradigm. Results showed that the time spent viewing both dynamic and static social vs. non-social stimuli was negatively associated with trait autism and significantly increased following intranasal oxytocin. For face stimuli, oxytocin primarily increased gaze towards the eyes of fearful expression faces but not for other face emotions. Overall, our findings demonstrate that oxytocin significantly shifts gaze preference towards social vs. non-social stimuli and to the eyes of fearful faces. Importantly, oxytocin appears generally to shift attention more towards salient social stimuli of particular relevance in the context of autism providing further support for its potential therapeutic use in autism-spectrum disorder.

## Introduction

Autistic individuals across cultures tend to show reduced interest in both dynamic and static social vs. non-social stimuli using eye-tracking measures^1^. We recently compared three such paradigms in children with autistic spectrum disorder (ASD) compared with typical developing (TD) controls^2^. The paradigm showing the greatest discriminatory accuracy was a dynamic task simultaneously contrasting social stimuli (individuals dancing) with geometric patterns and was also associated with symptom severity, particularly in relation to social dysfunction^2,3^. Two other paradigms, which discriminated ASD from TD children, were one comparing biological with non-biological motion using simultaneous point-light images and a static task where subjects view a toy/object alone (non-social) vs. the same toy-or object being played with by a person (social)^2^. However, the extent to which these same tasks are sensitive to autistic traits in healthy adults and influenced by potential therapeutic interventions is not established.

Our ability to recognize individuals and their facial expressions is of key importance for social behavior and interaction and ASD is associated with marked impairments in face-emotion recognition^4^, as well as social anxiety, depression^5–7^ and schizophrenia^8^. Previous eye-tracking studies have demonstrated that facial emotion recognition relies on a triangular visual search pattern, including eye-gaze towards the eyes as well as nose and mouth regions, although there is some degree of individual specificity^9^. In ASD and many other psychiatric disorders, the patterns of visual scanning of faces are abnormal with the most notable difference tending to be reduced time scanning the eyes and other salient regions, such as mouth and nose in comparison with other, less informative, face regions^1,10–12^. Thus, there is considerable interest in developing therapeutic approaches for normalizing eye-gaze patterns towards both social vs. non-social stimuli and salient facial cues in the context of ASD.

The hypothalamic neuropeptide oxytocin (OXT) has been reported to promote increased eye-gaze towards social stimuli^13,14^ and improve face-emotion recognition in healthy humans^15^ and may be a promising therapeutic candidate in the context of ASD^16^. While no studies to date have investigated the effects of intranasal OXT on the social vs. non-social task paradigms that are sensitive to ASD, a number of eye-tracking studies have reported effects on eye-gaze towards faces in both monkeys and humans, although results have been inconsistent. In humans, an initial study demonstrated increased gaze towards the eye region, but not the nose and mouth or cheeks and forehead, of statically presented neutral expression faces^17^. Other studies using dynamically presented faces have reported increased eye-gaze towards neutral expression faces with a marginal trend towards effects being maintained when faces changed to a happy expression and decreased when they changed to an angry one^18^ and increased eye-gaze across dynamic expressions of sadness, happiness, pain or fear^19^. Another study also reported that OXT increased the frequency of saccades from the mouth towards the eye region in angry, happy and fearful expression faces, with saccade frequency also being associated with amygdala responses^20^. In rhesus monkeys, OXT increased the number of fixations on the eyes relative to those on the mouth region when viewing faces of conspecifics, irrespective of whether neutral or threat expressions were displayed^21^. Similarly, in marmosets OXT also increased gaze time to the eyes relative to other face regions, and this effect could be prevented using an OXT receptor antagonist^22^. Importantly, OXT administration has also been reported to increase viewing of the eye region during viewing of neutral expression faces in autistic individuals required to make gender or gaze-direction decisions (fixation duration)^23^ and also during real-time social interactions in natural settings in both healthy control and autistic individuals (number of fixations per second)^24^.

However, some studies have failed to find significant effects of OXT on eye-gaze^25–27^. An early study with 47 male subjects found no effect of OXT on fixation time spent on either whole face or eye or mouth regions of static emotional faces (happy, sad, angry, and fearful) with different intensities although did find that OXT increased the recognition accuracy for fearful faces^25^. A subsequent study also found no effect of OXT in women on viewing the eyes relative to the rest of the face in static angry, fearful, happy, and neutral expression faces despite finding increased neural responses to either fearful or happy and angry faces, although data were from only 14 subjects^27^. Another more recent detailed study reported that while OXT marginally increased recognition speed, but not accuracy, for different static emotional faces (happy, angry, fear, sad, disgust and surprise) it did not increase the proportion of time spent viewing the eye region during the task^26^.

Thus, while a number of studies have reported that intranasal OXT increases the amount or proportion of time spent gazing at the eye region of faces others have failed to find effects and therefore further replication is important. Additionally, it is unclear whether there is an influence of specific emotional expressions. Furthermore, studies on human subjects to date have only included Caucasian populations and there is increasing evidence for different patterns of gaze to faces in Asian populations, with a greater focus on the nose region in Asian subjects compared to the eye, and lesser extent mouth regions in Caucasian ones^28–31^.

In the current study, we have therefore used a randomized within-subject placebo-controlled paradigm to investigate the effects of intranasal OXT (24IU) on patterns of eye-gaze in five different tasks involving social vs. non-social dynamic and static stimuli and also towards salient regions of emotional faces (eyes, nose, and mouth). In view of the potential therapeutic relevance of observed effects to autism we also investigated associations between OXT-effects and trait autism and empathy measured by routinely used questionnaires, the autism-spectrum quotient (AQ^32^—which measures all autistic symptoms), the Social Responsiveness Scale (SRS^33^—which focuses specifically on social impairments), and the Interpersonal Responsivity Index (IRI^34^—which focusses on trait empathy). We hypothesized firstly that higher levels of autistic and empathic traits would be associated with reduced viewing of social relative to non-social stimuli, and of the eyes relative to other face regions across emotional faces, and that secondly OXT treatment would increase interest in social stimuli and the eyes.

## Materials and methods

### Participants

Forty healthy male adults (mean age ± sem = 20.8 ± 0.38) were recruited via university advertisement. Only male subjects were recruited due to the main focus on potential relevance to autism and because the majority of previous studies have also only used male subjects. In a within-subject design, each participant attended the experiment twice and with an interval of around 2 weeks between each treatment/session (mean ± sem = 14.86 ± 0.16 days). Subjects randomly received either OXT (24 IU OXT in water, 0.9% sodium chloride and glycerol supplied by the Sichuan Meike Pharmaceutical Company, China) or placebo (PLC containing all ingredients except for the neuropeptide and supplied by the same company) intranasal treatment and the order of receiving OXT or PLC was counterbalanced. Subject number was determined by an a priori power analysis (using G-power with 1 group, 4 measurements, *α* = 0.05 and a 0.5 correlation between repeated measures), which showed that for a within-subject design 36 subjects would achieve >80% power for a medium effect size both with ANOVAs (*F* = 0.25, partial eta squared—0.06) and post-hoc pairwise comparisons (Cohen’s *d* = 0.5). We therefore decided to recruit 40 subjects to compensate for potential subjects dropping out and data collection issues. Subjects were required to abstain from caffeine, alcohol, nicotine, or other psychoactive substances in the 24 h before each experiment, and in initial interviews self-reported absence of current or previous psychiatric illness, drug, or alcohol abuse. All participants had normal or corrected to normal vision. The study had full approval from the local ethics committee of the University of Electronic Science and Technology of China and procedures were in accordance with the latest revision of the declaration of Helsinki. The study was pre-registered at clinical trials.gov (Trial registration ID: NCT03293511; URL: https://clinicaltrials.gov/ct2/show/NCT03293511). In the study, subjects actually completed seven different tasks but data from the final two tasks (involving directed attention and empathy) will be reported separately. All subjects signed written informed consent and paid for participation (100RMB).

### Experimental procedure

In a double-blind placebo-controlled within-subject design, subjects self-administered the nasal sprays (24 IU, three puffs per nostril, each containing four IU of OXT or PLC) following a standardized protocol^35^. One experimenter was responsible for assigning subjects to receive OXT or PLC first using a computer-generated randomization and was not involved in conducting the experiment. All nasal spray bottles were covered by black tape so that neither the second experimenter responsible for supervising intranasal administration and running the tasks nor the subjects could recognize their contents. At the first visit before treatment administration, participants initially completed a set of Chinese version questionnaires to evaluate mood and personality traits both as a control for possible confounders due to any treatment group differences (State Trait Anxiety Inventory—STAI^36^, Childhood trauma questionnaire—CTQ^37,38^). The Positive and Negative Affect Schedule (PANAS^39^) was completed three times before treatment administration (t1) and immediately before (t2) and after the tasks (t3) in order to assess possible treatment effects on mood. For the assessment of associations with experimental findings in the two groups in relation to autistic traits and empathy Chinese versions of the Autism-Spectrum Quotient (AQ^32^), Social Responsivity Scale (SRS^33^) and Interpersonal Responsivity Index (IRI^34^) were used.

Full task details are provided in the Supplementary Section. In brief, task 1 involved differential attention to dynamic social (dancing individuals) and non-social (geometric patterns) images modified from Pierce et al.^3^ and similar to our previous experiment comparing ASD and TD children^2^ (see Supplementary Fig. S1). Task 2 was a static face-emotion (FE) processing task where participants viewed 24 emotional faces, including happy, angry, fear, and neutral expressions from the same three males and three females (in color and with a 400 × 500 pixel resolution and black background—see Supplementary Fig. S1). The faces used were from the Taiwanese facial expression image database (TFEID) (http://bml.ym.edu.tw/tfeid/index.php). A novel task (Task 3) involved subjects viewing pairs of static facial images of human and emoticon faces (HEF) with the same neutral, happy or angry expression, and the other an emoticon (HEF see Supplementary Fig. S1). The expectation here was that individuals with higher trait autism would show a preference for the emoticon version of the emotional face. For task 4, we adapted our previous paradigm (Kou et al.)^2^ for static visual attention (SVA). Pairs of static images were displayed, one showing a picture of an adult with a happy face playing with a toy/object while looking outwards towards the subject (i.e., sharing their enjoyment) and the other only including the same toy/object (see Supplementary Fig. S1). For Task 5, visual preference for biological motion (BM) was investigated using point-light displaying identical sized animate (walking human or cat) and inanimate (randomly moving point-light) videos^2,40^ (see Supplementary Fig. S1). A flow chart of the whole-experiment procedure is provided in the supplementary (see Supplementary Fig. S2) and a CONSORT flow chart (see Supplementary Fig. S3).

### Eye-tracking data collection

Eye movement data were recorded using an eye tracker (Tobii TX300, Tobii, Danderyd, Sweden), which employs infrared (IR) light‐emitting diodes and IR camera to measure corneal reflections and calculate eye‐gaze direction. All gaze data were recorded at 300 Hz sampling rate with a gaze accuracy of 0.4°. Recording and stimulus presentation were conducted using Tobii Pro Studio, E-prime 2.0 software, and E-Prime Extensions for Tobii (Psychology Software Tools, Pittsburgh, PA). All raw data with at least 80% gaze weight were analyzed using Tobii studio. The primary measure collected in the current study was total fixation duration, although additionally the number of fixations and duration of individual fixations were measured in order to help interpret, which were contributing to any observed significant changes in total gaze time. See supplementary for full details of each task.

### Statistical analyses

All statistical analyses were performed using SPSS 22 (SPSS Inc., Chicago, IL, USA). In tasks 1, 3 and 4, two-way repeated ANOVAs were performed with two within-subject factors (treatment—OXT, PLC) and areas of interest (AOI -two regions), and total fixation duration as the dependent variable. For the FE task (Task 2) a three-way ANOVA was performed with treatment, AOIs (eyes, nose and mouth, other parts of the face) and face-emotion (angry, happy, fear, and neutral) as factors, and the percentage of total time spent viewing each region compared to the time viewing the screen as the dependent variable. For the eye region, we used an AOI only including the left and right eye, and not the bridge of the nose in line with observed scanning patterns by subjects and with several other eye-tracking experiments comparing Chinese and Caucasian subjects^28,31^ (AOIs and typical viewing patterns are shown in Supplementary Fig S4). We used percentages for the relative amount of time spent viewing each region compared to the whole screen as the dependent variable similar to previous studies investigating effects of OXT. In task 5, the difference in total fixation duration for the social stimulus (walking human) minus the total fixation duration to the scrambled sequence was calculated and subtracted from that for the non-social stimulus (i.e., cat–scrambled sequence). The overall difference score for PLC was then compared with that for OXT using paired *t*-tests. Where significant main or interaction effects were observed in the different tasks for total fixation duration, additional analyses were performed with the total number of fixations (fixation count), or percentages of fixations on difference face regions (Task 2), and average individual fixation duration (fixation duration) as dependent variables to determine which were contributing to the observed changes in overall fixation duration. The results of these secondary analyses are given in the supplementary and show that for the majority of tasks the effects of OXT on total or percentage fixation durations were due to altered fixation counts. Bonferroni correction was applied to all post-hoc tests. Associations between total or percentage fixation durations and autistic and empathy traits (AQ, SRS, and IRI scores) were performed using Pearson correlation and permutation analysis (bootstrap = 10,000 using the method described by Baguely^41^ and Wilcox^42^ for non-independent samples) used to assess possible treatment group differences. To correct for multiple comparisons (three questionnaires), the threshold *p*-value was adjusted to *p* < 0.0167 for the correlation analyses (e.g., *p* = 0.05/3 = 0.0167).

## Results

### Demographic and questionnaire data and effects of treatment on mood and overall gaze

We performed independent *t*-tests to show that there were no significant differences in terms of mood and personality traits between subjects with the two different treatment orders (see Supplementary Table. S1). A two-way repeated ANOVA with 2 treatment × 3 time points for PANAS scores revealed no significant main or interaction treatment effects on positive or negative mood (all ps > 0.665).

For all five tasks, there were no significant difference in overall gaze time towards the screen between the treatment groups or tasks (on average, subjects were viewing the screen 97–98% of the time—see Supplementary Table S2). There was also no significant difference in time spent viewing the actual stimuli across treatments (Task 1: *t*_(1,39)_ = 0.771, *p* = 0.446; Task 2: *t*_(1,35)_ = 1.758, *p* = 0.088; Task 3: *t*_(1,35) _= 1.227, *p* = 0.228; Task 4: *t*_(1,35)_ = 1.183, *p* = 0.245; Task 5: *t*_(1,32) _= –0.542, *p* = 0.592). Thus, OXT did not affect the total time spent viewing the screen or the stimuli in each task.

### Effects of oxytocin in the dynamic social attention (DSA) task (Task 1)

**Fig. 1:**
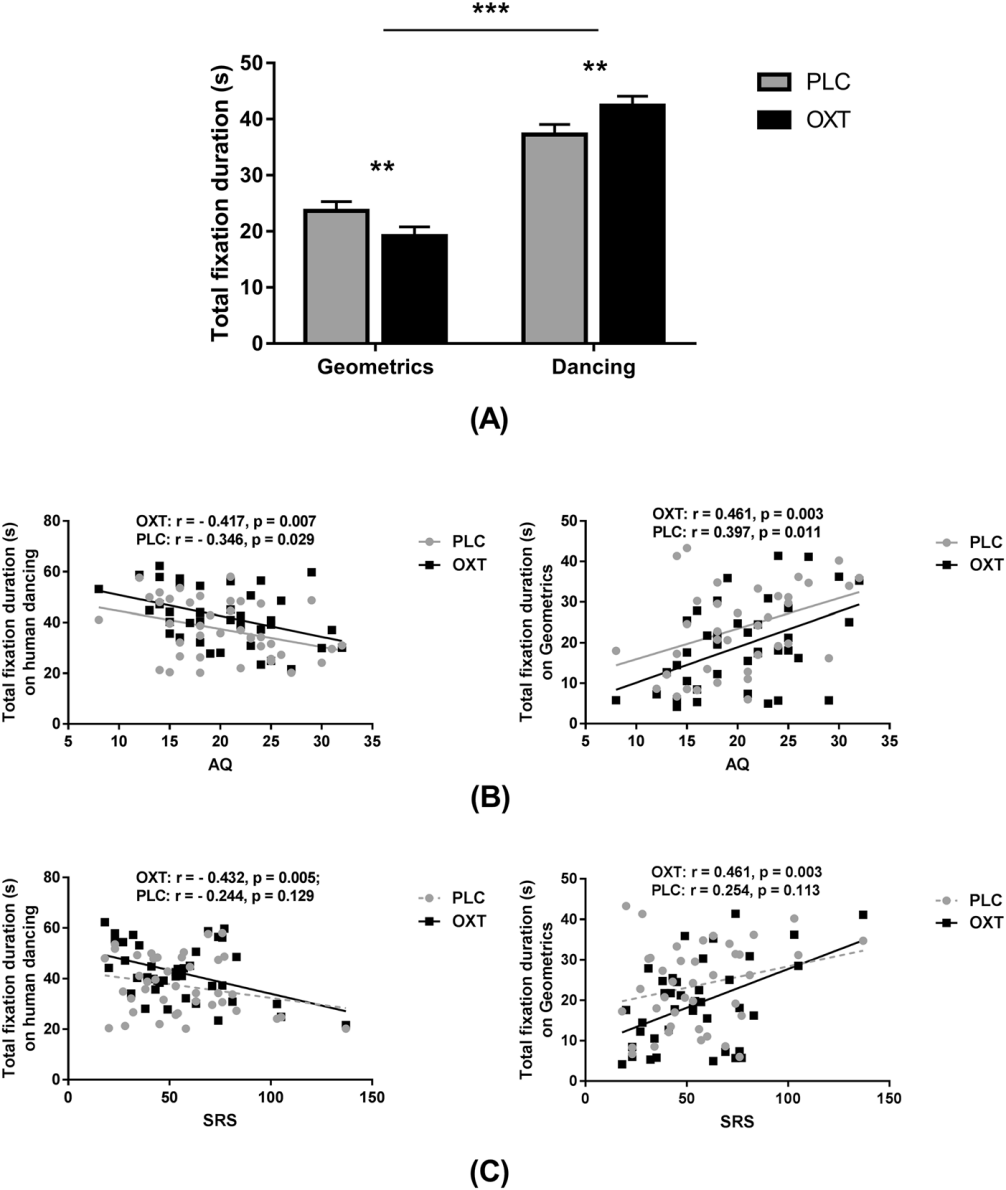
The effects of oxytocin (OXT) on eye-gaze towards dynamic social and geometric stimuli in the dynamic social attention (DSA) task—Task 1. **a** The effects OXT on mean total fixation duration for each type of stimulus in subjects following either OXT or placebo (PLC) treatment. Error bars represent SEM. **p < 0.01, *p < 0.05, OXT vs. PLC and Social vs. Geometric. **b**, **c** Correlation (Pearson) between scores on two autistic traits questionnaires and total fixation duration. AQ autism-spectrum quotient, *SRS* Social Responsiveness Scale.

A two-way repeated ANOVA with treatment and type (social vs. non-social AOIs) as within-subject factors and total fixation duration as the dependent variable revealed no main effect of treatment (*F*_(1, 39) _= 0.594, *p* = 0.446, partial *ƞ*^*2*^ = 0.015), but a main effect of type (*F*_(1, 39)_ = 39.258, *p* < 0.001, partial *ƞ*^*2*^ = 0.502) and a treatment x type interaction (*F*_(1, 39) _= 8.665, *p* = 0.005, partial *ƞ*^*2*^ = 0.182). A post-hoc Bonferonni-corrected analysis revealed that OXT treatment significantly increased the time spent looking at the social (person dancing) (*t*_(1, 39)_ = 2.923, *p* = 0.006, Cohen’s *d* = 0.470) and decreased that for the non-social (dynamic geometric) stimuli (*t*_(1, 39) _= –2.801, *p* = 0.008, Cohen’s *d* = 0.442) (see Fig. 1a).

### Relationship between autistic traits and eye-gaze in Task 1

For both OXT and PLC treatments AQ and SRS scores were negatively correlated with total fixation duration for the dynamic social stimuli (person dancing) (AQ: OXT: *r* = – 0.471, *p* = 0.007; PLC: *r* = –0.346, *p* = 0.029 but does not pass correction; SRS: OXT: *r* = – 0.432, *p* = 0.005; PLC: *r* = –0.244, *p* = 0.129), although the treatment difference was not significant (AQ: *p* = 0.585; SRS: *p* = 0.179). They were also positively correlated with total fixation duration for the dynamic non-social stimuli (geometric patterns) (AQ: OXT: *r* = 0.461, *p* = 0.003; PLC: *r* = 0.397, *p* = 0.011; SRS: OXT: *r* = 0.461, *p* = 0.003; PLC: *r* = 0.254, *p* = 0.113), although again the treatment difference was not significant (AQ: *p* = 0.666; SRS: *p* = 0.184) (see Fig. 1b, c). There were no significant correlations observed between total fixation duration and IRI scores (all rs < 0.158). Details of all correlations are given in Supplementary Table S3.

### Effects of oxytocin in the face-emotion processing (FE) task (Task 2)

**Fig. 2:**
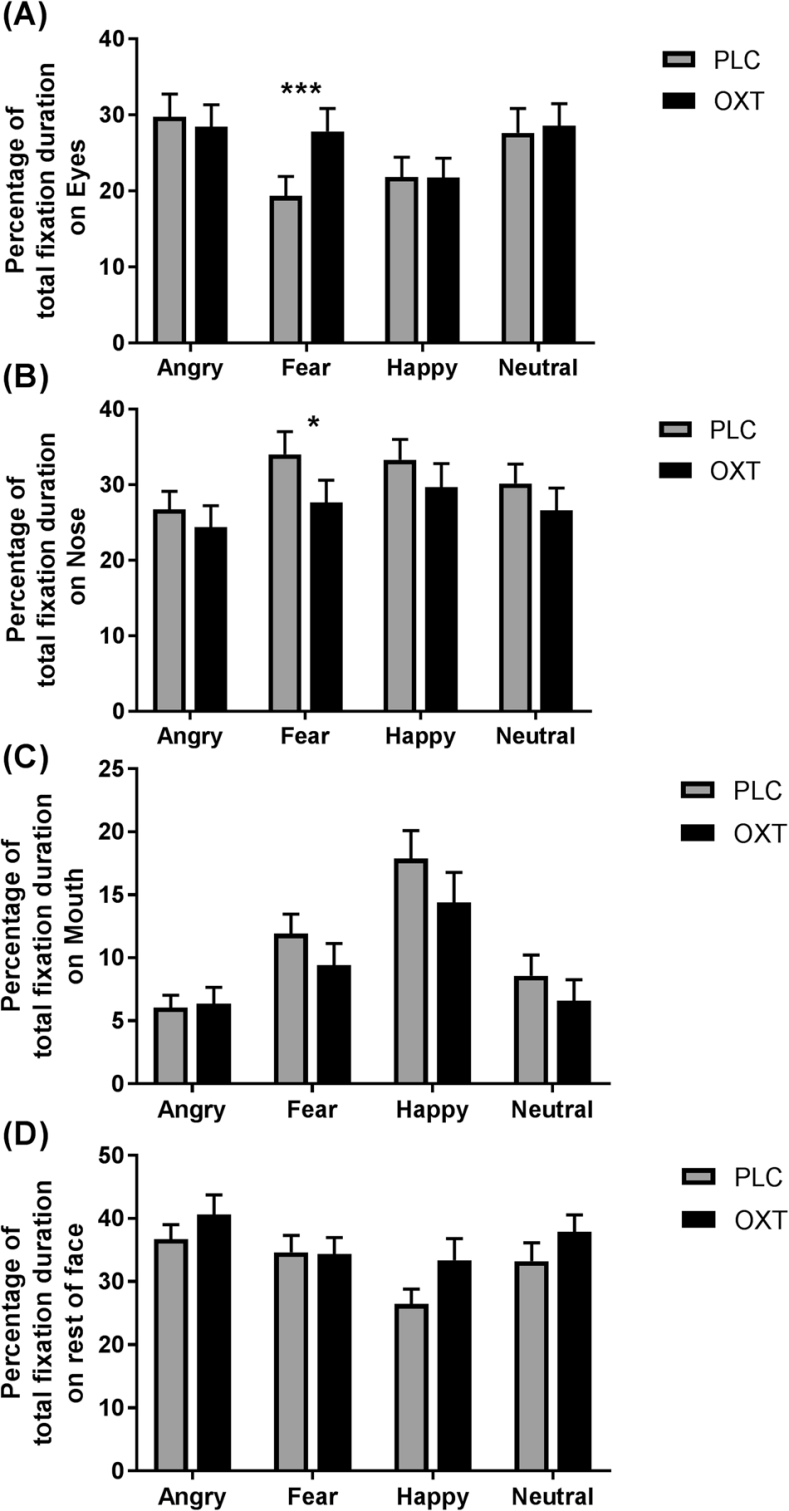
The effects of oxytocin (OXT) treatment on eye-gaze towards emotional faces - Task 2 (FE). Percentage of time viewing the eyes (**a**), nose (**b**), mouth (**c**) regions, and the rest of face (**d**) during the static face-emotion (angry, fear, happy, and neutral expressions) processing task. Mean percentage total fixation durations on different regions are relative to time spent viewing the screen during the presentation of faces. Error bars represent SEM. ***p<0.001, **p<0.01, *p<0.05, OXT vs. PLC.

Four subjects were excluded from analysis of the FE task due to technical problems with data collection, resulting in *n* = 36 subjects. A three-way ANOVA analysis was performed with treatment (OXT vs. PLC), face region (eyes, nose, mouth, other parts of the face—see Fig. 2) and face-emotion (angry, fear, happy, neutral) as factors. There was no main effect of face-emotion (*F*_(3, 105)_ = 0.597, *p* = 0.592) but a significant main effect of face region (*F*_(1, 35)_ = 22.287, *p* < 0.001), due to the mouth region being viewed less than the other three regions (ps < 0.001), and an emotion x region interaction (*F*_(3, 105)_ = 10.095, *p* < 0.001, partial *ƞ*^*2*^ = 0.224). Post-hoc Bonferroni corrected tests showed that the eyes were viewed less for happy than angry and neutral faces (ps < 0.012), the nose less for angry than fearful or happy faces (ps < 0.032). The mouth was viewed more for happy faces than all other emotions (ps < 0.001) and for fear more than angry and neutral faces (ps < 0.017), and the rest of the face was viewed less for happy than for angry and fearful faces (ps < 0.020. There was no significant main effect of treatment (*F*_(1, 35)_ = 0.099, *p* = 0.755) or treatment x emotion (*F*_(3, 105)_ = 1.763, *p* = 0.182) or treatment x region (*F*_(1, 35) _= 1.868, *p* = 0.157) interactions, but there was a significant treatment x region x emotion interaction (*F*_(3, 105) _= 2.154, *p* = 0.046, partial *ƞ*^*2*^ = 0.058). Post-hoc Bonferonni-corrected tests revealed that OXT increased the percentage of total fixation duration for the eyes of fearful faces (*t*_(1, 35) _= 3.567, *p* = 0.001, Cohen’s *d* = 0.601) and decreased it for the nose region (*t*_(1, 35) _= –2.487, *p* = 0.042, Cohen’s *d* = 0.415), but not for the mouth (*p* = 0.130) or other parts of the face (*p* = 0.799) (see Fig. 2 and Supplementary Fig S4 for examples of heat maps). Analysis of the proportion of fixation counts displayed produced similar findings (see supplementary).

### Relationship between autistic traits and eye-gaze in face-emotion task (Task 2)

Pearson correlation analyses were used to determine if autistic (AQ and SRS) or empathic (IRI) traits were significantly associated with the proportion of time viewing the different face regions across emotions or for specific emotions. For autistic traits, there was a significant positive correlation under OXT, but not PLC, for AQ scores for the nose region of happy expression faces (OXT—*r* = 0.420, p = 0.011; PLC—*r* = 0.037, *p* = 0. 829), while for SRS scores were positively correlated with viewing the nose region of neutral faces (OXT—*r* = 0.414, *p* = 0.012; PLC—*r* = –0.181, *p* = 0.290). In all cases, the correlation difference between OXT and PLC was significant (all ps < 0.006). Thus, higher autistic traits tended to be associated with a greater percentage of time viewing the nose region in happy and neutral expression faces under OXT. For trait empathy (IRI scores), there was a positive correlation with the percentage total fixation duration for the eyes of happy expression faces (OXT—*r* = 0.490, *p* = 0.002; PLC—*r* = 0.117, *p* = 0.495) following OXT but not PLC administration. There was a similar association for neutral (OXT—*r* = 0.364, *p* = 0.029; PLC—*r* = 0.051, *p* = 0.770) and angry faces (OXT—*r* = 0.331, *p* = 0.049; PLC—*r* = 0.038, *p* = 0.827), which did not pass correction. For the eyes of happy expression faces the correlation difference between OXT and PLC for IRI scores was significant (*p* = 0.007) and marginal for neutral (*p* = 0.05), and angry (*p* = 0.070) ones. Thus, OXT tended to increase the percentage of total fixation duration for the eyes of happy and to a lesser extent neutral and angry expression faces in individuals with higher empathy scores.

### Effects of oxytocin on gaze towards human and emoticon faces (HEF—Task 3)

**Fig. 3:**
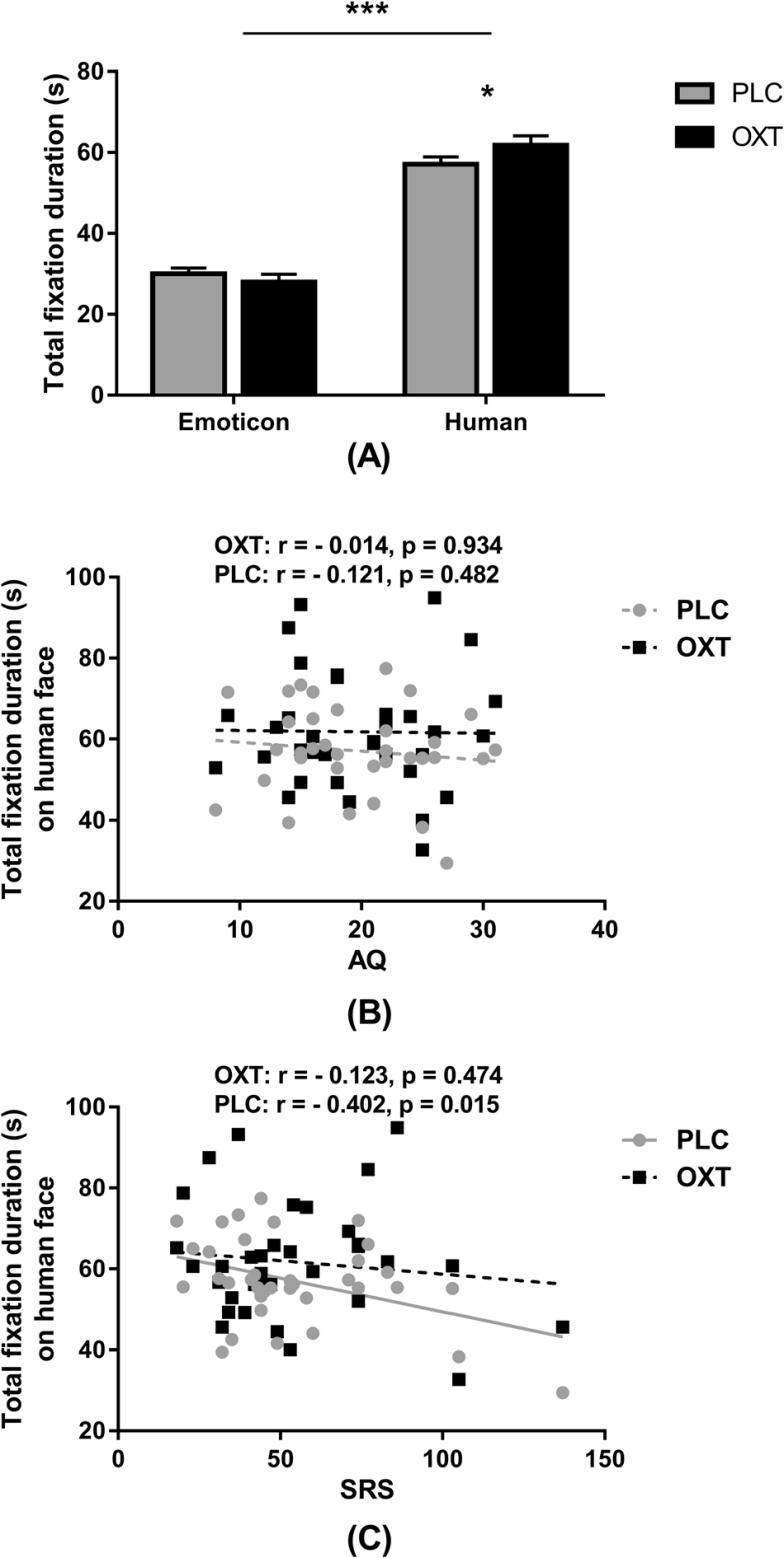
The effects of oxytocin (OXT) treatment on eye-gaze directed towards emotional human or emoticon faces—Task 3. **a** Mean total fixation durations for each type of stimulus in subjects following either OXT or placebo (PLC) treatment. Error bars represent SEM. **p < 0.01, *p < 0.05, OXT vs. PLC and Human faces vs. Emoticons. **b**, **c** Correlation (Pearson) between scores on autistic traits questionnaires and total fixation duration. AQ autism-spectrum quotient, SRS Social Responsiveness Scale.

Four subjects were excluded from analysis of the HEF task due to technical problems with data collection resulting in *n* = 36 subjects. Two-way ANOVA analysis revealed a significant effects of stimulus category (*F*_(1, 35) _= 106.178, *p* < 0.001, partial *ƞ*^*2*^ = 0.752) but not treatment (*F*_(1, 35)_ = 2.295, *p* = 0.139, partial *ƞ*^*2*^ = 0.062) on total fixation duration due to subjects spending a longer time looking at human faces across treatment conditions. There was also a significant x social category (*F*_(1, 35)_ = 4.10, *p* = 0.05, partial *ƞ*^*2*^ = 0.105) interaction. Post-hoc Bonferonni-corrected tests revealed that OXT significantly increased total fixation duration on the human face compared to PLC treatment (*t*_(1, 35)_ = 2.235, *p* = 0.032, Cohen’s *d* = 0.379) (see Fig. 3a).

### Relationship between autistic traits and eye-gaze in human and emoticon face task (Task 3)

The SRS score was significantly negatively associated with total fixation duration on the human face under PLC, but not OXT (OXT: *r* = –0.123, *p* = 0.474; PLC: *r* = –0.402, *p* = 0.015), but the treatment difference was not significant (*p* = 0.1). There were no significant correlations with AQ or IRI (all ps > 0.058, see Supplementary Table S3 and Fig. 3b, c).

### Effects of oxytocin in the static visual attention (SVA) task (Task 4)

**Fig. 4:**
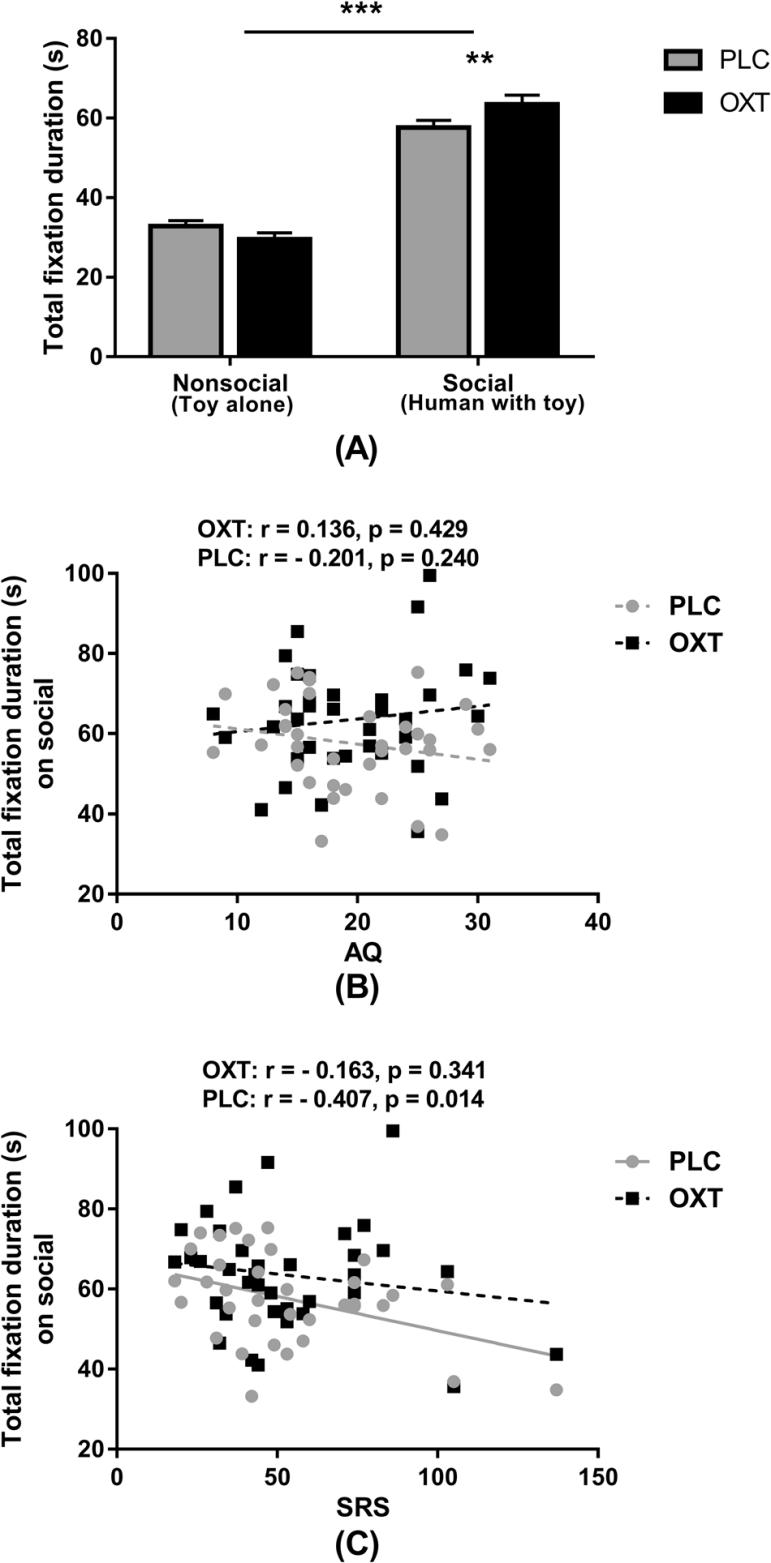
The effects of oxytocin (OXT) treatment on eye-gaze directed towards human with toy/object or toy/object during the static visual attention task—Task 4. **a** The effects OXT on mean total fixation duration for each type of stimulus in subjects following either OXT or placebo (PLC) treatment. Error bars represent SEM. **p < 0.01, *p < 0.05, OXT vs. PLC or human with toy/object or toy/object alone. **b**, **c** Correlation between scores on two autistic traits questionnaires and total fixation duration. AQ autism-spectrum quotient, SRS Social Responsiveness Scale.

Four subjects were excluded from analysis of this SVA task due to technical problems with data collection resulting in *n* = 36 subjects. A two-way ANOVA with treatment (OXT vs. PLC) and stimulus category (social—human with play object vs. non-social—play object alone) as factors investigated the effect of OXT on total fixation duration. There was no significant main effect of treatment (*F*_(1, 35)_ = 1.399, *p* = 0.245, partial *ƞ*^*2*^ = 0.038) but a main effect of category (*F*_(1, 35)_ = 129.102, *p* < 0.001, partial *ƞ*^*2*^ = 0.787) due to subjects showing a greater total fixation duration on the social category (person playing with toy/object) across treatments. There was also a significant treatment x category interaction (*F*_(1, 35)_ = 8.870, *p* = 0.005, partial *ƞ*^*2*^ = 0.202) and post-hoc Bonferonni-corrected tests revealed that OXT increased the total fixation duration on the social stimulus relative to PLC (*t*_(1, 35)_ = 2.951, *p* = 0.006, Cohen’s *d* = 0.499) (see Fig. 4a).

### Relationship between autistic traits and eye-gaze in the static visual attention task (Task 4)

Pearson correlation analyses revealed that the SRS score was negatively associated with total fixation duration on the social stimulus (human with toy/object) in the PLC but not the OXT condition (PLC: *r* = –0.407, *p* = 0.014; OXT: *r* = –0.163, *p* = 0.341) although the difference between the correlations was not significant (*p* = 0.1). No significant correlations with AQ or IRI were observed (all ps > 0.151) (see Supplementary Table S3 and Fig. 4b, c).

### Effects of oxytocin on gaze towards social vs. non-social biological motion (BM) (Task 5)

**Fig. 5:**
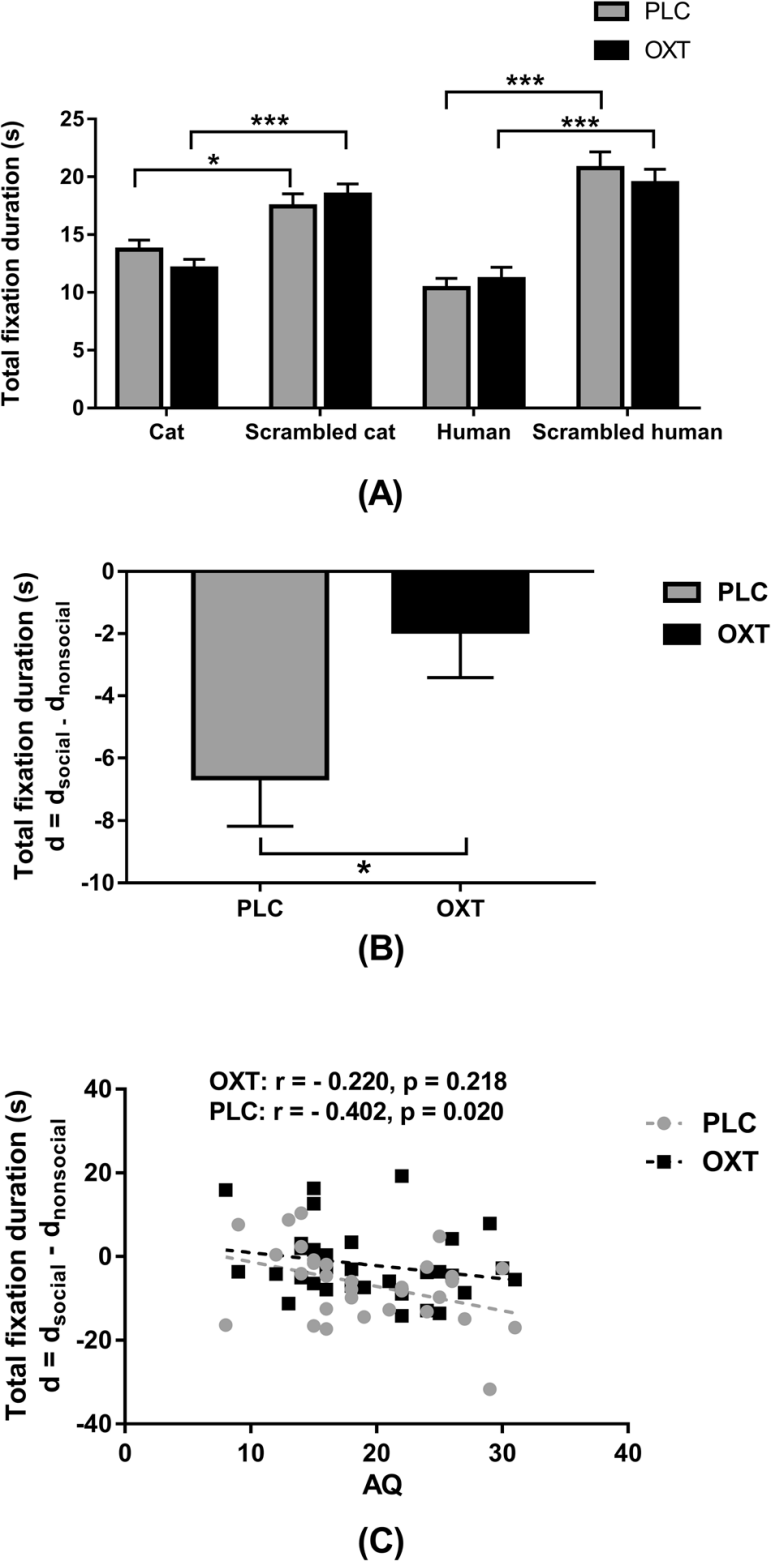
The effects of oxytocin (OXT) treatment on eye-gaze directed towards social (human walking) or non-social (cat walking) stimuli during the Biological motion task. **a** The effects OXT on total fixation duration for each type of stimulus in subjects following either OXT or placebo (PLC) treatment. Error bars represent SEM. **p < 0.01, *p < 0.05 for cat vs. scrambled or human vs. scrambled. **b** The effects of OXT on the difference in total fixation duration for human minus scrambled (social) and cat minus scrambled (non-social). Error bars represent SEM. **p < 0.01, *p < 0.05, OXT vs. PLC. **c** Correlation (Pearson) between scores on the autistic spectrum quotient (AQ) and differences in total fixation duration.

Four subjects were excluded from analysis of this BM task due to technical problems with data collection and three subjects due to presentation problems in their second session. Thus, data from *n* = 33 subjects were analyzed. Overall subjects showed greater total fixation duration to the scrambled stimuli compared to the cat or human ones (see Fig. 5a). To determine treatment effects on social vs. non-social biological motion we used difference score for total fixation duration as the dependent variable (*d*_nonsocial _= cat – scrambled Cat vs. *d*_social_ = human – scrambled human). A paired sample *t-*test revealed that OXT significantly decreased the difference between the total fixation duration on social vs. non-social biological motion (*t*_(1, 32)_ = 2.232, *p* = 0.033, Cohen’s *d* = 0.394) (see Fig. 5b). Analysis of fixation counts and individual fixation durations did not reveal which contributed to OXT-effects on total fixation duration in the BM task (see Supplementary Results).

### Relationship between autistic traits and eye towards social vs. non-social biological motion (Task 5)

Pearson correlation analyses revealed no significant correlations between questionnaires and total fixation durations during the biological motion task after correction for multiple comparisons (all ps > 0.020) (see Supplementary Table S3 and Fig. 5c).

## Discussion

The current study employed an eye-tracking approach in healthy adult male Chinese subjects to determine whether intranasal OXT increased gaze towards either dynamic or static social vs. non-social stimuli in four different tasks sensitive to autistic traits, and additionally towards the eye region of emotional faces. Our results demonstrate that OXT treatment increased the amount of time spent viewing social stimuli across all four different tasks. Viewing time in all four tasks showed negative associations with autistic traits (either AQ or SRS scores) in both PLC and OXT treatment conditions, suggesting that OXT was promoting increased interest in social stimuli equivalently across trait autism scores. For face emotions, OXT only increased the percentage of time viewing the eyes and decreased that for the nose for fearful expression faces. Thus, the effects of OXT on increasing time spent viewing the eyes appears to be highly emotion specific in this context. Viewing time for the eyes was also positively associated with trait empathy (IRI) for angry, happy, and neutral expression faces following OXT administration.

The dynamic social vs. geometric pattern paradigm (Task 1) has been shown to be one of the most robust for identifying children with ASD, as well as the severity of their social symptoms in both Caucasian^3,43^ and Chinese populations^2,44^. In the current study, we have confirmed this negative association between interest in the social stimuli and trait autism (AQ and SRS scores) in healthy adult subjects. For the other three tasks (tasks 3, 4, and 5) total fixation duration on the social stimuli were also significantly negatively associated with either AQ or SRS scores, supporting their utility as being sensitive to autistic symptoms. There was, however, no association within subjects between autism scores and the differences in total fixation duration between PLC and OXT treatments, suggesting that overall effects of OXT were similar across the range of autistic symptom magnitudes.

In the biological motion task, subjects tended to pay more attention towards the scrambled light point displays rather than either those showing a human or a cat, which is the opposite of the pattern usually reported in young children^2,45,46^. Possibly this reflects an age effect with greater interest exhibited by adults trying to interpret the scrambled images and, therefore, paying more attention to them. However, in the healthy adults we did find a negative correlation with AQ scores when we used difference scores between the total fixation duration for human/cat vs. scrambled stimuli, whereas in ASD children there were no associations with symptom severity.

Importantly, in the context of the primary objective of the current study, intranasal OXT significantly increased the total amount of time spent viewing the social stimuli in all four paradigms (both dynamic and static) with its effect appearing to be similar across subjects independent of their trait autism scores. A secondary analysis of fixation counts and individual fixation durations indicated that the effects of OXT result primarily from an increased number of fixations towards the social stimuli, although for the human vs. emoticon faces and biological motion tasks, changes in both numbers of fixations and individual fixation durations may have contributed.

For face expression stimuli, OXT only increased the proportion of time spent looking at the eyes of static fearful expression faces and correspondingly decreased that for viewing the nose region. This contrasts with some previous studies using static presentations reporting that OXT either increased the proportion of time viewing the eyes of neutral expression faces^17^ or did not influence it at all for neutral or emotional faces in males^25,26^ or females^27^, although the latter study included very few subjects. These discrepant findings may be due to several factors. Unlike most previous studies, we only measured viewing time for the eyes themselves and did not additionally include parts of the face between or above them. This reflected the typical gaze patterns of our subjects where the main focus was on the eyes themselves rather than regions between or above them. Indeed, the average proportion of time viewing the eyes in our study was approximately half of that reported in another study^26^, although this may reflect cultural differences since our Chinese subjects viewed the eyes and nose for similar amounts of time, whereas Caucasian subjects view the eyes more than all other regions^28–31,47^. Asian subjects appear to use a nose-centric scanning pattern during face recognition in contrast to Caucasians who use an eye-centric^28–31^. Another relevant contributory factor could be that the subjects in the current study viewed faces passively, whereas the previous study on male Caucasians reporting no effects of OXT used an active task paradigm where subjects were required to identify each different face-emotion^26^. Another study reporting no effect of OXT on eye-gaze also used task rather than passive viewing conditions where participants were required to recognize different intensity emotional faces as quickly possible^25^. Previous findings using dynamic presentations of face emotions on gaze towards the eyes have used a variety of different approaches but produced somewhat inconsistent results. One study found OXT significantly increased the time spent viewing the eye region^19^, while another only found a marginal effect of OXT on happy faces^18^, and one reported no effect of OXT at all^25^. For static faces, one study has reported OXT increased time spent viewing the eyes of neutral expression faces^17^, and another that it increased gaze shift upwards to the eye region across different emotions and related to brain amygdala activity^20^. However, two other studies have found no effect of OXT on time spent viewing the eyes^26,27^. Thus, findings have generally tended to be inconsistent, and at this stage it is still unclear whether OXT may have different effects on statically compared to dynamically presented face stimuli or during task-related compared to passive viewing conditions.

Our finding that OXT primarily influenced gaze towards the eyes and away from the nose of fearful faces resonates with a number of previous observations on its behavioral and neural effects. A meta-analysis of the effects of OXT on face-emotion recognition reported effects for fearful, angry, and happy faces with the effect size being much larger for fearful faces (0.591)^15^, and increased fear face recognition has also been reported in schizophrenia patients^48^. The eye region is of particular importance for the recognition of fearful faces and the amygdala appears to be a key region for controlling this^49^. The amygdala is one of the primary neural targets for functional effects of OXT and a number of studies have reported that OXT alters amygdala responses to both fearful faces across cultures^27,50–52^ and subliminal presentation of the eyes of fearful faces^50^. Another study has reported that the frequency of shifts in gaze (saccades) upwards from the mouth to the eyes of fearful faces is increased following OXT, and that amygdala responses to emotional faces are associated with such upward gaze shifts^20^. Interestingly, accuracy in recognizing face identity, gender and emotion is associated with such upward shifts of gaze from lower regions of the face towards the eyes^53^, Other studies have also reported that OXT can increase attentional orientation^54^ and self-reported empathy in response to fearful faces^19^. However, it should be noted that in the latter study self-reported empathy was negatively associated with time spent looking at the eyes^19^. One study reporting positive effects of OXT on the recognition accuracy failed to find any association with proportion of time spent viewing the eyes^25^ while another reporting marginal effects of OXT on reducing recognition time across face emotions also failed to find any association with proportion of time spent viewing the eye region^26^. Thus, the relationship between the effects of OXT on viewing the eyes and recognition of, and behavioral responses to, face emotions remains unclear.

While intranasal OXT consistently decreases amygdala responses to threatening faces in males it has the opposite effect in females^51,55,56^, and an early study reported that increased amygdala responses to fear in a small number of female subjects were not influenced by gaze towards the eyes^27^. Similarly, another study has found that increased amygdala responses to emotional scenes are not associated with patterns of eye-gaze in women^51^. Thus, while OXT may increase interest in threatening faces/scenes in males it may not do so in females. Future larger scale studies are therefore needed to investigate the possibility of sex differences in effects of OXT on patterns of eye-gaze towards emotional faces.

We did not find evidence for a significant association between trait autism scores and time spent viewing the eyes, even though individuals with ASD do tend to show this^10,57^. A previous study has reported a similar lack of association between trait autism and time spent viewing the eyes in healthy subjects^58^. However, a meta-analysis of the degree of impaired recognition of emotional faces in ASD has shown that fear faces have the largest effect size^59^. There was also a positive correlation between autistic traits and viewing the nose region in both happy and neutral expression faces under OXT, indicating that it may influence Chinese subjects with higher autistic traits to look more at the nose region of positive valence faces. Following OXT but not PLC administration we did observe a positive association with empathy (IRI) scores and the proportion of time spent viewing the eyes of happy expression faces and also to a lesser extent neutral and angry ones. Thus, in healthy subjects, time spent viewing the eyes may be more strongly associated with empathic rather than autistic traits following OXT administration.

Overall, our findings from the four social vs. non-social tasks are in line with the hypothesis that OXT promotes greater attention generally towards social stimuli. However, the findings from the face-emotion task showing that it biases attention towards the eyes relative to the nose of fearful faces, support the social salience hypothesis, which proposes that OXT functions primarily to enhance attention towards social cues that are of particular importance for an individual, with detection of threat being highly relevant for survival^60^.

There are several limitations in the current study. Firstly, in line with other studies investigating eye-gaze effects of OXT, only male subjects^19,20,52,61,62^ were included given the focus on relevance to autism. As discussed above, it is possible that there are sex differences in the effects of OXT on both eye-gaze and amygdala responses in the context of fear faces and a number of studies have reported other sex-dependent effects of OXT (see refs. ^13,48,60,63^). Future studies will need to recruit both male and female subjects to address this. Secondly, we used a fixed rather than randomized order of task presentation, although importantly we found significant effects of OXT across all five tasks.

In summary, our findings show for the first time that intranasal OXT enhances visual preference for dynamic and social vs. non-social stimuli in four different tasks sensitive to autistic traits. Additionally, they demonstrate that OXT promotes gaze towards the eye region and away from the nose region of static fearful, but not other face expressions. Overall, our findings therefore provide further support for the potential of OXT in therapeutic interventions for autism through shifting attention more towards social stimuli. While the effects of OXT on increased gaze towards the eyes were only significant for fearful expressions, recognition of fearful faces is the most impaired in ASD^59^. This might still suggest OXT could be beneficial therapeutically by helping to improve interpretation of social threat cues, although some caution may be needed since ASD is often co-morbid with anxiety disorders^64^. Further studies on clinical populations are required to better assess if OXT more generally promotes gaze towards the eyes and improves emotion recognition.

## Supplementary information

Supplementary Material

CONSORT Checklist

## References

[1] Chita-Tegmark M (2016). Social attention in ASD: a review and meta-analysis of eye-tracking studies. Res. Dev. Disabil..

[2] Kou J (2019). Comparison of three different eye-tracking tasks for distinguishing autistic from typically developing children and autistic symptom severity. Autism Res..

[3] Pierce K (2016). Eye tracking reveals abnormal visual preference for geometric images as an early biomarker of an autism spectrum disorder subtype associated with increased symptom severity. Biol. Psychiatry.

[4] Weigelt S, Koldewyn K, Kanwisher N (2012). Face identity recognition in autism spectrum disorders: a review of behavioral studies. Neurosci. Biobehav. Rev..

[5] Dalili MN, Penton-Voak IS, Harmer CJ, Munafò MR (2015). Meta-analysis of emotion recognition deficits in major depressive disorder. Psychological Med..

[6] Demenescu LR, Kortekaas R, den Boer JA, Aleman A (2010). Impaired attribution of emotion to facial expressions in anxiety and major depression. PLoS ONE.

[7] Kohler CG, Hoffman LJ, Eastman LB, Healey K, Moberg PJ (2011). Facial emotion perception in depression and bipolar disorder: a quantitative review. Psychiatry Res..

[8] Kohler CG, Walker JB, Martin EA, Healey KM, Moberg PJ (2009). Facial emotion perception in schizophrenia: a meta-analytic review. Schizophrenia Bull..

[9] Mehoudar E, Arizpe J, Baker CI, Yovel G (2014). Faces in the eye of the beholder: Unique and stable eye scanning patterns of individual observers. J. Vis..

[10] Black MH (2017). Mechanisms of facial emotion recognition in autism spectrum disorders: Insights from eye tracking and electroencephalography. Neurosci. Biobehav. Rev..

[11] Loughland CM, Williams LM, Gordon E (2002). Schizophrenia and affective disorder show different visual scanning behavior for faces: a trait versus state-based distinction?. Biol. Psychiatry.

[12] Yi L (2013). Abnormality in face scanning by children with autism spectrum disorder is limited to the eye region: evidence from multi-method analyses of eye tracking data. J. Vis..

[13] Eckstein M (2019). Oxytocin increases eye-gaze towards novel social and non-social stimuli. Soc. Neurosci..

[14] Putnam PT, Roman JM, Zimmerman PE, Gothard KM (2016). Oxytocin enhances gaze-following responses to videos of natural social behavior in adult male rhesus monkeys. Psychoneuroendocrinology.

[15] Shahrestani S, Kemp AH, Guastella AJ (2013). The impact of a single administration of intranasal oxytocin on the recognition of basic emotions in humans: a meta-analysis. Neuropsychopharmacology.

[16] Kendrick, K. M., Guastella, A. J., & Becker, B. in *Behavioral Pharmacology of Neuropeptides: Oxytocin.* 321–348 (Springer, Cham, 2017).

[17] Guastella AJ, Mitchell PB, Dadds MR (2008). Oxytocin increases gaze to the eye region of human faces. Biol. Psychiatry.

[18] Domes G, Steiner A, Porges SW, Heinrichs M (2013). Oxytocin differentially modulates eye gaze to naturalistic social signals of happiness and anger. Psychoneuroendocrinology.

[19] Hubble K (2017). Oxytocin increases attention to the eyes and selectively enhances self-reported affective empathy for fear. Neuropsychologia.

[20] Gamer M, Zurowski B, Büchel C (2010). Different amygdala subregions mediate valence-related and attentional effects of oxytocin in humans. Proc. Natl Acad. Sci..

[21] Dal Monte O, Noble P, Costa VD, Averbeck BB (2014). Oxytocin enhances attention to the eye region in rhesus monkeys. Front. Neurosci..

[22] Kotani M (2017). An eye tracking system for monitoring face scanning patterns reveals the enhancing effect of oxytocin on eye contact in common marmosets. Psychoneuroendocrinology.

[23] Andari E (2010). Promoting social behavior with oxytocin in high-functioning autism spectrum disorders. Proc. Natl Acad. Sci..

[24] Auyeung B (2015). Oxytocin increases eye contact during a real-time, naturalistic social interaction in males with and without autism. Transl. Psychiatry.

[25] Lischke A (2012). Intranasal oxytocin enhances emotion recognition from dynamic facial expressions and leaves eye-gaze unaffected. Psychoneuroendocrinology.

[26] Hubble K (2017). Oxytocin reduces face processing time but leaves recognition accuracy and eye-gaze unaffected. J. Int. Neuropsychological. Soc..

[27] Domes G (2010). Effects of intranasal oxytocin on emotional face processing in women. Psychoneuroendocrinology.

[28] Fu G, Hu CS, Wang Q, Quinn PC, Lee K (2012). Adults scan own-and other-race faces differently. PLoS ONE.

[29] Kelly DJ, Miellet S, Caldara R (2010). Culture shapes eye movements for visually homogeneous objects. Front. Psychol..

[30] Miellet S, Vizioli L, He L, Zhou X, Caldara R (2013). Mapping face recognition information use across cultures. Front. Psychol..

[31] Blais C, Jack RE, Scheepers C, Fiset D, Caldara R (2009). Culture shapes how we look at faces. PLoS ONE.

[32] Lau WYP (2013). Psychometric properties of the Chinese version of the Autism Spectrum Quotient (AQ). Res. Dev. Disabil..

[33] Gau SSF, Liu LT, Wu YY, Chiu YN, Tsai WC (2013). Psychometric properties of the Chinese version of the social responsiveness scale. Res. Autism Spectr. Disord..

[34] Davis MH (1980). A multidimensional approach to individual differences in empathy. Cat. Sel. Doc. Psychol..

[35] Guastella AJ (2013). Recommendations for the standardisation of oxytocin nasal administration and guidelines for its reporting in human research. Psychoneuroendocrinology.

[36] Shek DT (1993). The Chinese version of the State-Trait Anxiety Inventory: Its relationship to different measures of psychological well-being. J. Clin. Psychol..

[37] Bernstein DP (2003). Development and validation of a brief screening version of the Childhood Trauma Questionnaire. Child Abus. Negl..

[38] Zhao X, Zhang Y, Longfei LI, Zhou Y (2005). Evaluation on reliability and validity of Chinese version of childhood trauma questionnaire. Chin. J. Tissue Eng. Res..

[39] Watson D, Clark LA, Tellegen A (1988). Development and validation of brief measures of positive and negative affect: the PANAS scales. J. Personal. Soc. Psychol..

[40] Rutherford MD, Troje NF (2012). IQ predicts biological motion perception in autism spectrum disorders. J. Autism Dev. Disord..

[41] Baguley T (2012). Serious Stats: A Guide to Advanced Statistics for the Behavioral Sciences.

[42] Wilcox RR (2016). Comparing dependent robust correlations. Br. J. Math. Stat. Psychol..

[43] Moore A (2018). The geometric preference subtype in ASD: identifying a consistent, early-emerging phenomenon through eye tracking. Mol. Autism.

[44] Shi L (2015). Different visual preference patterns in response to simple and complex dynamic social stimuli in preschool-aged children with autism spectrum disorders. PLoS ONE.

[45] Annaz D, Campbell R, Coleman M, Milne E, Swettenham J (2012). Young children with autism spectrum disorder do not preferentially attend to biological motion. J. Autism Dev. Disord..

[46] Blake R, Turner LM, Smoski MJ, Pozdol SL, Stone WL (2003). Visual recognition of biological motion is impaired in children with autism. Psychological Sci..

[47] Scheller E, Büchel C, Gamer M (2012). Diagnostic features of emotional expressions are processed preferentially. PLoS ONE.

[48] Fischer-Shofty M, Shamay-Tsoory SG, Levkovitz Y (2013). Characterization of the effects of oxytocin on fear recognition in patients with schizophrenia and in healthy controls. Front. Neurosci..

[49] Adolphs R (2008). Fear, faces, and the hu*man amygdala*. Curr. Opin. Neurobiol..

[50] Kanat M (2015). Oxytocin modulates amygdala reactivity to masked fearful eyes. Neuropsychopharmacology.

[51] Lieberz, J. et al. (2019). Kinetics of oxytocin effects on amygdala and striatal reactivity vary between women and men. *Neuropsychopharmacology*. 10.1038/s41386-019-0582-649 10.1038/s41386-019-0582-6 PMC7235226 31785587

[52] Kruppa JA (2019). Neural modulation of social reinforcement learning by intranasal oxytocin in male adults with high-functioning autism spectrum disorder: a randomized trial. Neuropsychopharmacology.

[53] Peterson MF, Eckstein MP (2012). Looking just below the eyes is optimal across face recognition tasks. Proc. Natl Acad. Sci..

[54] Tollenaar MS, Chatzimanoli M, van der Wee NJ, Putman P (2013). Enhanced orienting of attention in response to emotional gaze cues after oxytocin administration in healthy young men. Psychoneuroendocrinology.

[55] Luo L (2017). Sex-dependent neural effect of oxytocin during subliminal processing of negative emotion faces. Neuroimage.

[56] Lischke A, Herpertz SC, Berger C, Domes G, Gamer M (2017). Divergent effects of oxytocin on (para-) limbic reactivity to emotional and neutral scenes in females with and without borderline personality disorder. Soc. Cogn. Affect. Neurosci..

[57] Tanaka JW, Sung A (2016). The “eye avoidance” hypothesis of autism face processing. J. Autism Dev. Disord..

[58] Davis J (2017). Social and attention‐to‐detail subclusters of autistic traits differentially predict looking at eyes and face identity recognition ability. Br. J. Psychol..

[59] Uljarevic M, Hamilton A (2013). Recognition of emotions in autism: a formal meta-analysis. J. Autism Dev. Disord..

[60] Shamay-Tsoory SG, Abu-Akel A (2016). The social salience hypothesis of oxytocin. Biol. Psychiatry.

[61] Schumacher S (2018). Does trait anxiety influence effects of oxytocin on eye-blink startle reactivity? A randomized, double-blind, placebo-controlled crossover study. PLoS ONE.

[62] Xu X, Li J, Chen Z, Kendrick KM, Becker B (2019). Oxytocin reduces top-down control of attention by increasing bottom-up attention allocation to social but not non-social stimuli–a randomized controlled trial. Psychoneuroendocrinology.

[63] Gao S (2016). Oxytocin, the peptide that bonds the sexes also divides them. Proc. Natl Acad. Sci..

[64] van Steensel FJ, Bogels SM, Perrin S (2011). Anxiety disorders in children and adolescents with autistic spectrum disorders: A meta-analysis. Clin. Child Fam. Psychol. Rev..

