# Supplementary material for: Oxytocin biases eye-gaze to dynamic and static social images and the eyes of fearful faces: associations with trait autism
Source: Transl Psychiatry. 2020 May 12;10:142. doi: 10.1038/s41398-020-0830-x (PMC7217872; doi:10.1038/s41398-020-0830-x)
Supplement: Supplementary file 1 — Supplementary Material [file 41398_2020_830_MOESM1_ESM.doc]

**Supplementary Information**

**Oxytocin biases eye-gaze to dynamic and static social images and the eyes of fearful faces: associations with trait autism**

Jiao Le*, Juan Kou*, Weihua Zhao, Meina Fu, Yingying Zhang, Benjamin Becker, Keith M Kendrick

**Supplementary methods**

**Task details**

For the experimental tasks, subjects were seated on a comfortable chair about 65 cm from a 23-inch computer screen (resolution 1920 × 1080 pixels, refresh rate 60 Hz), where stimuli were presented and instructed to view passively each stimulus. The current experiment involved seven tasks and here we report findings from the first five of them. The tasks were presented twice to the subjects in the same sequence (once following PLC and once following OXT given in a random order).

In the dynamic social attention (DSA) task (task 1), the two categories of stimuli (social vs geometric) were presented simultaneously on the left or right of the center of the screen with positions being counter balanced. Both pairs of dynamic stimuli were 72 seconds video clips with the social stimuli consisted of Chinese adults dancing alone (5 females and 5 males) while non-social stimuli included complex dynamic geometric shapes (see Figure.S1). A total of thirty-six pairs of stimuli were presented. To avoid possible confounds from subjects simply staring at one position on the screen all the time, we divided each 72s video clip into two parts (i.e. 36s) with the position of two stimuli being switched after the first 36 seconds during experiment. In accordance with previous studies using this task (Kou et al., 2019; Peirce et al, 2016) pairs of stimuli were presented in a continuous stream without intervals. At the beginning of the task subjects viewed a fixation cross in the center of the screen for 3 s before the stimuli were presented.

For the face emotion (FE) task (Task 2) each face was presented 2 seconds in a randomized order with a jittered 1500ms fixation interval (1-2.5s) between each face where a cross was displayed in the center of the screen. The same presentation timing was used in task 3 (Human face versus emoticon - HEF) with the position of the emoticon and face stimuli being randomized (i.e. left vs right).

In the static visual attention task (SVA) (Task 4) 20 pairs of stimuli (5 males and 5 females) were each shown for 3 seconds. To avoid any potential bias due to specific toys/objects 10 different pairings of adults and play objects were used (burr puzzle, kongming lock, airplane, Rubik's cube, 3DS, seven-piece puzzle, yoyo, house model, shuttlecock, ball). The position of the two types of stimuli on the left and right side was counterbalanced and the average size of the toys/objects in the paired images did not vary significantly. There was a jittered interval (mean = 3 s) between each pair of stimuli where a fixation cross was displayed in the center of screen.

For the biological motion (BM) task (Task 5) on the left and right side of the screen, animate (human or cat) stimuli *vs*. control inanimate (scrambled versions of the human and cat stimuli) were displayed simultaneously (8 pairs of stimuli with a 10 s presentation duration), and the position of the two types of stimuli on the left and right side was counterbalanced. There was a jittered interval (mean=3 s) between each pair of stimuli where a fixation cross was displayed in the center of screen.

**Eye tracking and data**

A Tobii TX300 Binocular Eye Tracker was used together with Tobii Studio software (version 3.4.8 Tobii, Stockholm, Sweden) to record visual attention and E-prime linked with the Tobii software used for stimulus presentation. The Tobii system used an I-VT fixation filter. The mean of the right and left eyes was used to calculate fixation. The eye-tracking monitor (TFT-LCD; 23", 1920×1080) had a refresh rate of 60 Hz. Brightness was 100 % and a five-point calibration procedure was used at the beginning of each experiment. The Tobii five-point calibration program was used and calibration was considered to be successful when both eyes had good mapping on all five test positions. When the error at one or more target locations was greater than 0.5 degrees, the calibration procedure was repeated at those target locations.

In the DSA task (Task 1), two same sized areas of interest (AOI, 574 × 431 pixels) were drawn for both the social and nonsocial stimuli. In the FE processing task (Task 2), four different sized AOIs were used: two eye regions (180 × 100 pixels each), nose region (250 × 130 pixels), mouth region (300 × 130 pixels) and whole face region (400 × 500 pixels) for each face expression. For the HEF task (Task 3) the two AOIs used were the human face and the emoticon face. For the SVA task (Task 4) and the BM task (task 5) the same approach was used as in task 1 with two identical size AOIs for the two presentation images.

**Supplementary Results**

**Analysis of fixation counts and individual fixation durations in Task 1 (DSA)**

A 2-way ANOVA analysis of fixation counts (treatment and category – social vs non-social as factors) revealed a significant main effect of stimulus type [F *_(1, 39)_* = 98.459, *p* < 0.001, partial *ƞ^2^* = 0.716] and a treatment x type interaction [F *_(1, 39)_* = 6.753, *p* = 0.013, partial *ƞ^2^* = 0.148]. Post-hoc Bonferonni corrected tests revealed that OXT significantly increased number of fixation counts on the social stimulus [*t _(1, 39)_* = 2.202, *p* = 0.034, Cohen’s *d* = 0.348]. For mean duration of individual fixations there was also a significant main effect of type [*F _(1, 39)_* = 10.257, *p* = 0.003, partial *ƞ^2^* = 0.208] and a treatment x type interaction [*F _(1, 39)_* = 4.208, *p* = 0.047, partial *ƞ^2^* = 0.097] suggesting that OXT reduced fixation durations to the non-social geometric stimuli, although post-hoc tests were not significant (non-social; t = - 1.618, *p* =0.114; social t – 0.342, *p* = 0.734). Thus, the OXT-evoked increase in time spent viewing the dynamic social stimuli in this task was contributed to mainly by increasing the number of fixations towards them.

**Analysis of fixation counts proportion and individual fixation durations in Task 2 (FE)**

For percentage of fixation counts 3-way ANOVAs (treatment, face emotion and face region as factors) revealed similar results as the percentage of total fixation duration. There were no main effects of treatment (*F* _(1, 35)_ = 0.373, *p* = 0.545) or of face emotion (*F* _(3, 105)_ = 0.103, *p* =0.913) but a significant main effect of face region (*F* _(1, 35)_ = 31.123, *p* < 0.003, partial ƞ2 = 0.471). The main effect of face region was due to subjects spending a proportionately smaller percentage of fixation counts on the mouth regions compared to eyes, nose and rest face region (all *ps* < 0.001) proportionately more on the rest of face compared to the eye (*p* = 0.007), nose (*p* = 0.003) and mouth region (*p* < 0.001) across treatments. There was no significant treatment x emotion interaction (*F* _(1, 35)_ = 0.361, *p* = 0.703) but there were significant treatment x region (*F* _(1, 35)_ = 3.812, *p* = 0.022, partial *ƞ2* = 0.098) and emotion x region (*F* _(3, 105)_ = 14.471, *p* < 0.001, partial *ƞ2* = 0.293) interactions. Post-hoc Bonferonni corrected tests revealed that treatment x region interaction was due to a significant reduction in the proportion of fixation counts on the nose (*p* = 0.02) and mouth regions (*p* = 0.023). The three-way interaction with treatment x emotion x region (*F* _(3, 105)_ = 2.265, *p* = 0.037, partial *ƞ2* = 0.061) was also significant. Post-hoc Bonferonni corrected t-test indicated OXT significantly increased proportion of fixation counts on the eye region of fearful faces (*p* = 0.001) and decreased them on the nose (*p* < 0.001). OXT also decreased the percentage of fixation counts on the neutral mouth (*p* = 0.007).

We conducted same analysis with individual fixation duration as dependent variable. The results indicated no main effect of treatment (*F* _(1, 35)_ = 3.563, *p* = 0.068) or of face emotion (*F* _(3, 105)_ = 1.319, *p* =0.274) but a significant main effect of face region (*F* _(1, 35)_ = 13.695, *p* < 0.001, partial *ƞ^2^* = 0.300). The main effect of face region was due to subjects spending an increased individual fixation duration on the nose compared to eye region (*p* = 0.004) and mouth region (*p* < 0.001) Subjects also showed longer individual fixation durations on the rest of the face compared to the eye (*p* = 0.028) and mouth regions (*p* = 0.002) across treatments. There were no significant interaction effects of treatment x emotion (*F* _(3, 105)_ = 0.664, *p* = 0.549) or of treatment x region (*F* _(1, 35)_ = 1.456, *p* = 0.239) or treatment x emotion x region (*F* _(3, 105)_ = 1.212, *p* = 0.305). There was a significant and emotion x region (*F* _(3, 105)_ = 2.728, *p* = 0.018, partial *ƞ^2^* = 0.079) interaction.

Thus overall, the OXT-induced increase in the percentage of total fixation time towards the eyes relative to nose and mouth was primarily contributed to by increased numbers of fixations rather than their durations.

**Analysis of fixation counts and individual fixation durations in Task 3 (HEF)**

Two way ANOVAs for fixation counts and individual fixation durations in the HEF task revealed only a main effect of social category [*F _(1, 35)_* = 187.89, *p* < 0.001, partial *ƞ^2^* = 0.843] for fixation counts due to subjects showing more fixations on the human faces compared to the emoticons, but there was no main effect of treatment or treatment x category interaction. There were no significant effects for individual fixation durations (all ps > 0.074). Thus, it is not clear whether treatment effects on tfd were contributed to by altered fixation counts or fixation durations.

**Analysis of fixation counts and individual fixation durations in Task 4 (SVA)**

A two-way ANOVA for the number of fixation counts in the SVA task (treatment and category – social versus non-social) revealed a significant main effect of social category [F *_(1, 35)_* = 139.217, *p* < 0.001, partial *ƞ^2^* = 0.847] and a treatment x social category [F *_(1, 35)_* = 6.588, *p* = 0.015, partial *ƞ^2^* = 0.158] interaction. Post-hoc tests revealed that OXT significantly enhanced the number of fixations for the social category compared to PLC [*t _(1, 35)_* = -2.229, *p* = 0.032, Cohen’s *d* = 0.372]. For individual fixation duration there was no main effect of treatment [F *_(1, 35)_* = 3.206, *p* = 0.082, partial *ƞ^2^* = 0.084] or social category [F *_(1, 35)_* = 3.138, *p* = 0.085, partial *ƞ^2^* = 0.082] or treatment x category interaction [F *_(1, 35)_* = 2.161, *p* = 0.150, partial *ƞ^2^* = 0.058]. Thus, the effect of OXT on increasing total fixation durations towards the social stimulus was mainly due to an increase in the number of fixations.

**Analysis of fixation counts and individual fixation durations in Task 5 (BM)**

A paired t-test comparing the difference in fixation counts and individual fixation durations for (human minus scrambled) minus (cat minus scrambled) following OXT and PLC treatment revealed no significant results [fixation counts: *t _(1, 32)_* = 1.128, *p* = 0.268, Cohen’s *d* = 0.196; fixation durations: *t _(1, 32)_* = - 1.587, *p* = 0.122, Cohen’s *d* = 0.275]. Thus, it is unclear which contributed to the altered pattern of total fixation durations for social biological motion following OXT.

**Supplementary tables and figures**

**Table S1** Participant demographic and questionnaire data in individuals receiving OXT or PLC treatment first (means and SEMs).

| Measurements | First taking OXT  (n = 20) | First taking PLC  (n = 20) | t | P |
| --- | --- | --- | --- | --- |
| Age | 21(0.60) | 21(0.48) | -0.394 | 0.696 |
| SRS | 59(6.79) | 51(4.31) | 0.957 | 0.344 |
| AQ | 20(1.12) | 21(1.36) | -0.823 | 0.416 |
| SAI | 36(2.02) | 37(2.00) | -0.228 | 0.821 |
| TAI | 41(1.85) | 41(1.77) | 0.313 | 0.756 |
| CTQ | 27(1.24) | 27(1.17) | -0.147 | 0.884 |
| IRI | 48(1.50) | 48(1.83) | 0.169 | 0.867 |

Group differences were analyzed by t test. SRS: Social Responsiveness Scale; AQ: Autism-Spectrum Quotient; SAI, TAI: State and Trait Anxiety Inventory; CTQ: Childhood Trauma Questionnaire; IRI: Interpersonal responsivity index.

**Table S2** Total fixation durations (means and SEMs) and the percentage of total time fixed on screen during oxytocin (OT) and placebo (PLC) treatments across the five different tasks

| Task | treatment | Task duration (s) | Total fixation time on screen (s) | Percentage of time fixed on screen |
| --- | --- | --- | --- | --- |
| 1  (n = 36) | OT  PLC | 72 | 69(0.09)  70(0.15) | 97.1(0.13)  96.8(0.21) |
| 2  (n = 36) | OT  PLC | 48 | 47(0.27)  47(0.50) | 98.1(0.57)  97.8(1.04) |
| 3  (n = 36) | OT  PLC | 120 | 116(0.83)  117(0.54) | 96.6(0.69)  97.1(0.45) |
| 4  (n = 36) | OT  PLC | 120 | 118(0.52)  117(0.67) | 98.6(0.44)  97.8(0.56) |
| 5  (n = 33) | OT  PLC | 80 | 78(0.70)  78(0.74) | 97.2(0.88)  97.5(0.93) |

Task 1: Dynamic social (Dancing) vs non-social (Geometric) stimuli; Task 2: Human emotional faces; Task 3: Human and emoticon faces (HEF); Task 4: Static visual attention (SVA) for toy/object alone vs human plus toy/object; Task 5: biological motion; for human versus scrambled and cat versus scrambled. s = seconds

**Table S3** Correlations between three autistic traits related questionnaires and four tasks under OXT or PLC treatment and OXT – PLC condition (Pearson correlations and significances)

| Task | Dependent variable | Stimuli | Treatment | AQ | SRS | IRI |
| --- | --- | --- | --- | --- | --- | --- |
| 1  (n = 40) | tfd | Dancing | OXT | -.417^**^ | -.432^**^ | .086 |
|  |  |  | PLC | -.346^*^ | -.244 | -.135 |
|  |  | Geometric | OXT | .461^**^ | .461^**^ | .028 |
|  |  |  | PLC | .397^**^ | .254 | .158 |
| 3  (n = 36) | tfd | Human face | OXT | -.014 | -.123 | .200 |
|  |  |  | PLC | -.121 | -.402^**^ | .089 |
|  |  | Emoticon | OXT | -.038 | .078 | -.083 |
|  |  |  | PLC | .261 | .304 | -.182 |
| 4  (n = 36) | tfd | Human & toy | OXT | .136 | -.163 | .008 |
|  |  |  | PLC | -.201 | -.407^**^ | .035 |
|  |  | Toy only | OXT | -.074 | .020 | .125 |
|  |  |  | PLC | .124 | .133 | .103 |
| 5  (n = 33) | tfd | Diff_Bio | OXT | -.220 | -.281 | -.095 |
|  |  |  | PLC | -.402^*^ | -.289 | -.128 |

Correlations were analyzed by Pearson correlation;

**. Correlation is significant corrected at the 0.0167 level (2-tailed).

*. Correlation is significant uncorrected at the 0.05 level (2-tailed).

Task 1: Dancing vs Dynamic Geometric; Task 3: human and emoticon faces (HEF); Task 4: static visual attention (SVA); Task 5: biological motion; tfd: total fixation duration; Diff_Bio: d_nonsocial_ (tfd_cat_ – tfd_scrambledCat_) - d_social_ (tfd_human_ – tfd_scrambledHuman_); AQ: Autism-Spectrum Quotient; SRS: Social Responsiveness Scale; IRI: Interpersonal responsivity index. Correlations between the differences scores between OXT and PLC treatments were also calculated but none were significant after correction and so the data is not presented in this table.

**
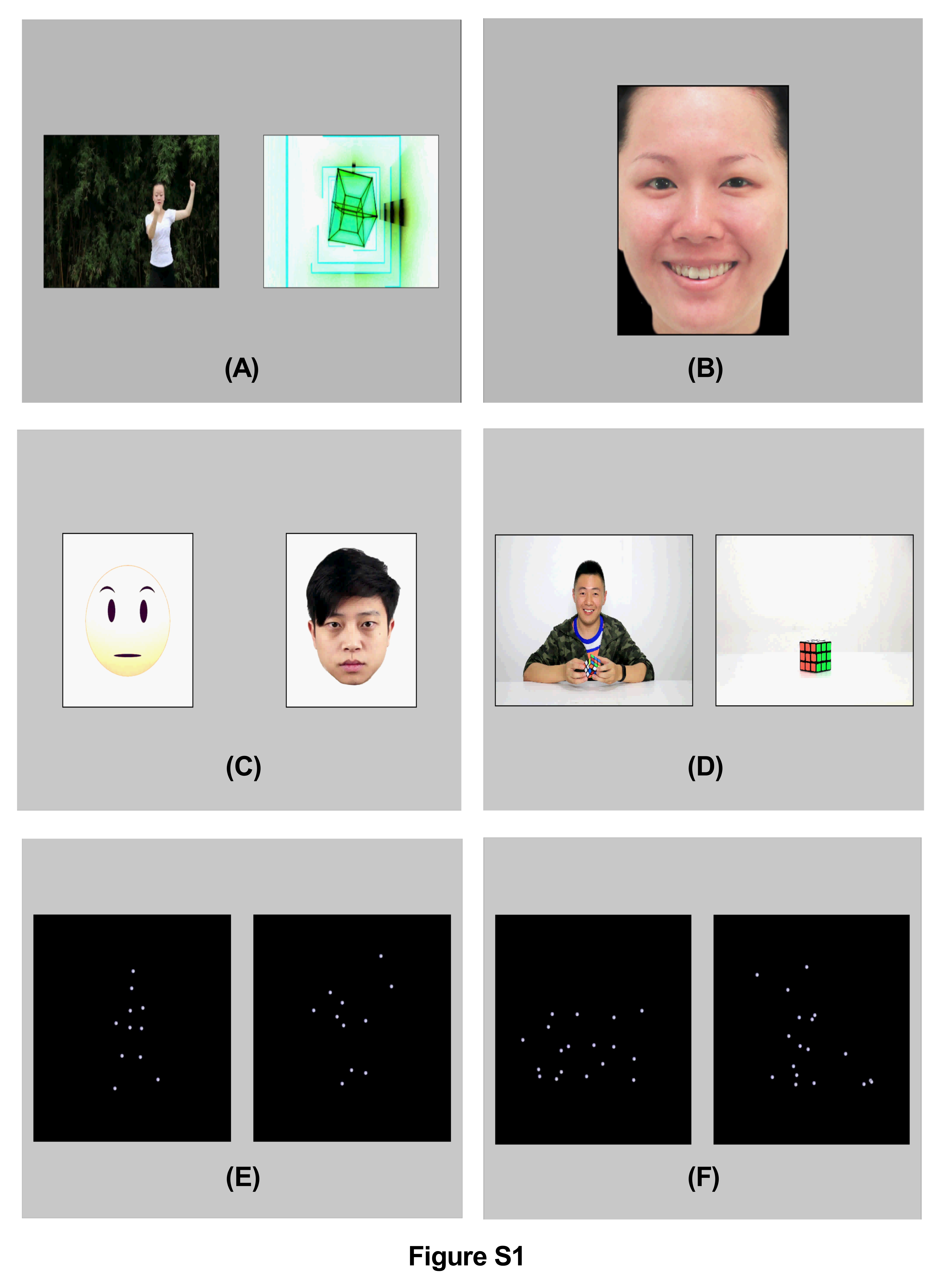
Figure S1.** Examples of stimuli in the five eye-tracking tasks (A) dynamic social (dancing individuals) vs dynamic geometric stimuli – Task 1; (B) static emotional face processing – Task 2; (C) emotional human vs emoticon faces – Task 3; (D) human with toy/object vs toy/object alone – Task 4; (E) dynamic point light display of human walking vs scrambled (F) cat walking vs scrambled


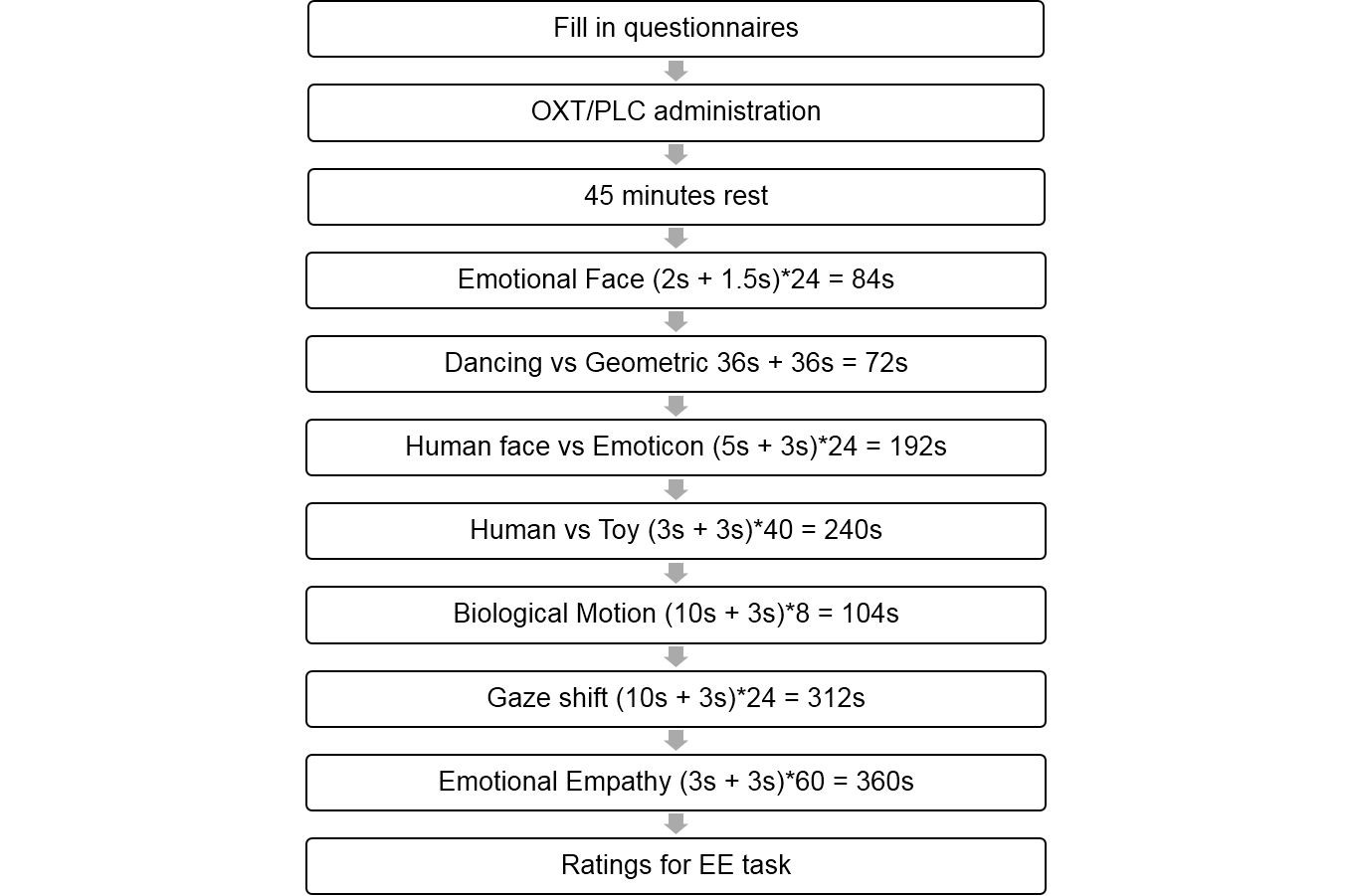


**Figure S2.** Flow chart of experiment with the fixed order of the eye tracking task. S (seconds); EE (emotional empathy).


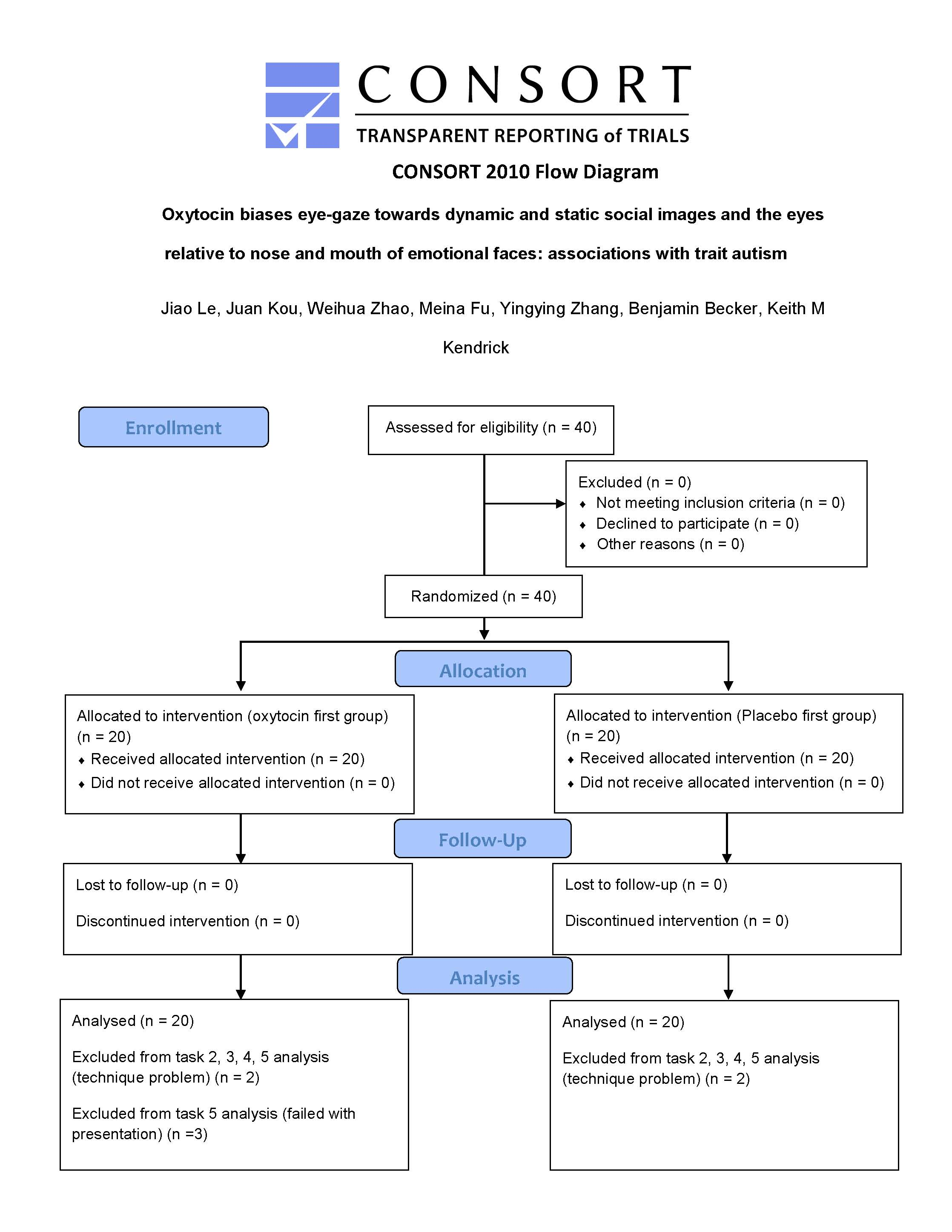


**Figure S3** CONSORT Flow Diagram

**Figure S4.** Examples of heat maps illustrating effects of oxytocin (OXT) treatment on eye gaze on individual fearful faces during the static face emotion (FE) processing task. Warm colors indicate greater amounts of time spent viewing specific regions and heat maps

 are averaged across all 36 subjects. The areas of interest for the eyes, nose and mouth regions are denoted by black lines.
